# The Correlation Between Autistic Childhood Disorders and the Development of Anxiety and Depression in Adults: A Systematic Review

**DOI:** 10.7759/cureus.30093

**Published:** 2022-10-09

**Authors:** Akinkunmi Kilanko, Crystal Obi-Azuike, Ngozi Adaralegbe, Chioma Eze-Njoku, Alexsandra Urhi, Chukwudi Agbor, Oghenetega E Ayisire, Victor C Eche, Fareena Soomro, Garima Kaur, Funmilola Babalola, Oluwabukola C Oyeleye-Adegbite, Bialo Aladum, Hakeem A Popoola, Gibson O Anugwom

**Affiliations:** 1 Behavioral Sciences, La Sierra University, Riverside, USA; 2 Psychiatry and Behavioral Sciences, University of Medicine and Health Sciences, New York City, USA; 3 Psychiatry and Behavioral Sciences, University of Connecticut, Waterbury, USA; 4 Pediatrics, Atrium Health Navicient the Medical Center, Macon, USA; 5 Psychiatry, Federal Neuro Psychiatric Hospital, Lagos, NGA; 6 Psychiatry, Tees, Esk and Wear Valleys NHS Trust, Scarborough, GBR; 7 Psychiatry, University of South Wales, Pontypridd, GBR; 8 Psychiatry, University of Port Harcourt, Port Harcourt, NGA; 9 Psychiatry and Behavioral Sciences, Isra University, Sindh, PAK; 10 Psychiatry, All Indian Institute of Medical Sciences Rishikesh, Rishikesh, IND; 11 Epidemiology and Public Health, Texas Department of State Health Services, San Antonio, USA; 12 Public Health, Texas A&M University, College Station, USA; 13 Psychiatry, Ascension Borgess Hospital, Kalamazoo, USA; 14 Internal Medicine, Windsor University School of Medicine, Bassettere, KNA; 15 Psychiatry and Behavioral Sciences, Baylor College of Medicine, Houston, USA

**Keywords:** correlation, autistic childhood disorders, anxiety, depression, autism spectrum disorder and anxiety disorder

## Abstract

Children with autism spectrum disorder significantly suffer from other mental conditions, including anxiety and depression, compared to the general population. This continues to have a significant effect till adulthood. This study aimed at determining if there is a correlation between autism disorder in childhood and the development of anxiety and depression in adulthood and if behavioral therapy for children with this disorder reduces the likelihood of developing anxiety and depression as an adult.

Three major databases were searched: EMBASE, Google Scholar, and PubMed, using specific search terms. Studies were selected according to population, exposure, comparison, condition or outcome(s) of interest, study design, and context. Overall, there are five articles relevant to this systematic review synthesis; all were observational studies. Our study shows psychiatric disorders like anxiety and depression could be related to autism spectrum disorder and early behavioral intervention could be beneficial and reduce the need for anxiety and depression medication.

## Introduction and background

Autism spectrum disorder (ASD) is a neurodevelopmental disorder characterized by difficulties with social communication and the presence of restricted interests and repetitive behaviors [1]. Autism was first recognized as a diagnostic entity in 1943 by Kamer [2].

This disorder affects approximately one in 100 children worldwide [3]. Increased levels of anxiety and depression are seen in children with autism compared to the general population, which may translate into adulthood [4-6]. Adults with ASD may also be at increased risk of developing anxiety and depression [5,7].

To clarify the frequency and progression of anxiety and depression in the population of adults that were diagnosed with autism in childhood, our study aims to systematically review and summarize current research works on the correlation between childhood autism and adulthood depression and anxiety.

## Review

Methodology

We searched three major databases - Embase, PubMed (MEDLINE), and Google Scholar using specified search terms. Search terms on the Medline database are ((autism) AND (depression)) AND (anxiety) AND (adults). The search terms used for Embase are autism in childhood” OR ((“autism/exp OR autism”) AND (“childhood”/exp OR childhood)) AND (“development of anxiety” OR ((“development”/exp OR development) AND of AND (“anxiety”/exp OR anxiety)) AND (“depression”/exp OR depression). The search terms used for Google “Scholar are (autism in kids) AND (anxiety and depression) AND (anxiety in adults) AND (depression in adults) advanced with the exact phrase “autism anxiety depression.” A search was done on PROSPERO and Cochrane library to check if a review on this topic has been done to prevent duplication, and none was found. Also, the review protocol has been submitted to PROSPERO.

Study selection

Studies were selected according to the following criteria: population, exposure, comparison, condition or outcome(s) of interest, study design, and context.

Inclusion Criteria

These include original research articles that examine and report the relationship between autism and anxiety and depression in adulthood, report the effect of Autistic Behavioral Therapy on an autistic kid and the likelihood of such therapy reducing anxiety and depression.

Exclusion Criteria

Literature and systematic review studies, editorials, commentaries, original articles irrelevant to autism, and outcomes not focused on anxiety and depression.

Data collection and study assessment

All the authors independently reviewed the abstracts of all the articles identified. The articles adopted were based on the inclusion criteria. The adopted papers were then screened, and a spreadsheet was then created to include all the proposed articles to be used for this research work. All authors were involved in the final selection process. 

Data synthesis and analysis

Quantitative and qualitative studies based on original research examining the correlation between ASD in children and the development of anxiety and depression as adults, the possibility of behavioral therapy in children reducing the likelihood of developing anxiety and depression, and the use of psychiatric medications. The data synthesis was done in a clear and detailed descriptive summary of the studies via tabulating. The quantitative data were extracted using Microsoft Word. All the identified concepts and themes were arranged and grouped to synthesize significant themes. All authors were responsible for reviewing and discussing major identified themes in the study.

Results

**Figure 1:**
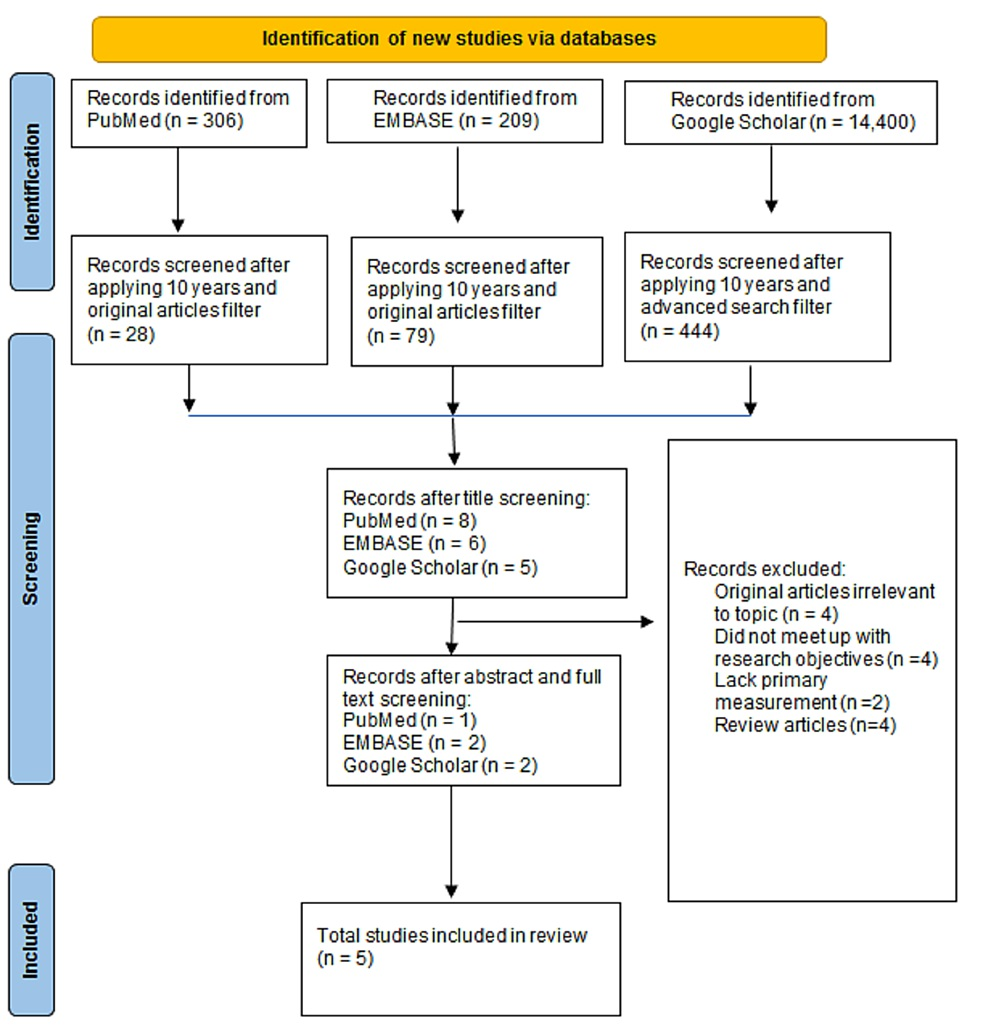
A PRISMA flow diagram showing the literature selection process.

Using the Preferred Reporting Items for Systematic Review [8], our data search returned 515 articles from PubMed and Embase, while Google Scholar turned 14,400 in the initial search before we initiated an advanced search with specific terms related to our study, thereby yielding 551 articles (Figure 1). We screened for relevance by title, abstract, language (English), date (2012 to 2022), relevance to study objectives, and article type (original articles). The screening resulted in 19 articles. After a detailed and analytical review, four articles were excluded based on irrelevance, four on not meeting the research objectives, two on lacking primary measurement, and four on review articles.

Discussion 

The DSM-IV diagnoses broadly classify anxiety disorder as generalized anxiety disorder, obsessive-compulsive disorder, separation anxiety disorder, and social phobia while it classifies depression disorder into dysthymic disorder and major depressive disorder [9]. Very little existing literature has shown an association between ASD and co-occurring disorders like mood and anxiety disorders. The study conducted by Gotham et al. determined the association between verbal IQ and gender with growth in anxiety and depressive symptoms in the ASD population [4]. The findings of the study only revealed a positive correlation between Verbal IQ and anxiety but not the development of depressive symptoms in this population [4]. It was observed that individuals on the spectrum who were less verbal had higher internalizing symptoms over time tested and these findings aligned with the findings of Hallet et al. [10]. Internalizing symptoms in the ASD population remained present all through the adolescence period of development [4]. This makes it further challenging for a diagnosis of depression to be made early in individuals with ASD.

Verbal IQ has been linked to socio-economic status (SES), with special reference to maternal education [11]. Lower SES has also been shown to contribute significantly to the development of behavioral symptoms in individuals with ASD. Mayes et al. showed a sufficient relationship between the level of psychosocial stress in the participants to the behavioral problems observed in the study [12]. This was attributed to the impaired ability of the parents to tolerate the stress of the child and was found to be significantly higher in children of professional parents compared to children of non-professional parents [12]. According to the studies reviewed in this paper, lack of financial resources also accounted for a general lack of implementation of effective coping and behavioral techniques, mainly arising from reduced access to high-quality clinical services for their children [11,12].

With respect to gender, this factor was not a strong determinant of the development of either anxiety or depression in children with ASD, as their symptoms varied throughout the school-age period as well as their adolescent years. The study conducted by Gotham et al. revealed that females with a diagnosis of ASD showed an increase in the anxiety and depressive symptoms investigated compared with the male gender [4]. This increase in depressive and anxiety symptoms was only evident in the adolescent period and not during their young adulthood development [4]. Higher levels of anxiety or depressive symptoms were noted more in school-aged boys compared to girls with ASD [4]. A well-noted feature of ASD is their tendency to insist on and maintain similar routines and this characteristic is said to be related to an increasing state of stress and thus the development of anxiety symptoms [13]. According to Hofvander et al., individuals with ASD are known to have a restricted repertoire of appropriate coping mechanisms, and this further predisposes them to the development of anxiety symptoms [14]. A higher frequency of major depressive disorder was observed in the study by Hofvander et al. [14] and this was attributed to a slightly higher median age (28 years) in the study, again this is not an uncommon finding, given their tendency to insist on sameness. Persons with ASD are more likely to have one form of personality disorder or the other, more so an obsessive-compulsive personality disorder. A significant level of psychosocial impairment was noted in the ASD sample assayed despite their normal or high level of intelligence [14].

The number of original studies regarding the relationship between ASD in childhood and the development of anxiety and depression in adulthood is severely limited. Understanding the rates of comorbid conditions within those with ASD may aid in the development of screening tools and effective therapies. Research has found substantial levels of depression, anxiety, and suicide attempts among adults with ASD, which were attributed to social anxiety, which was attributed to social difficulty, and perceived burdensomeness [15,16]. As seen in this review, factors such as gender, IQ, and psychosocial stress contributed to the development of comorbid mood disturbances in those with ASD. Awareness of the variables that contribute to the development of anxiety and depression in adulthood among children with ASD can assist in the creation of interventional and preventative strategies. Overall, there are five articles relevant to this systematic review synthesis and all were observational studies (Table 1).

**Table 1:** Characteristics and summary of findings from the included studies

Author/Year	Study Design	Population/Sample size/ demographics	Summary
Gotham K et al. 2015 [4]	Observational study	165 participants (n = 109 with ASD; n = 56 with non-spectrum disorder)	Anxiety was positively associated with verbal intelligent quotient (IQ). Anxiety and depressive symptoms were greater in those with Autism Spectrum Disorder (ASD) than non-spectrum subjects. Female gender associated with greater increases over time in anxiety and depressive symptoms. Males with ASD have higher levels of depressive symptoms in school-age that continue into young adulthood
Nimmo-Smith V et al. 2020 [5]	Observational study	Population sample: 221,694>18 years of whom 4049 were diagnosed with ASD	Anxiety disorders were diagnosed in 20.1% of adults with ASD compared with 8.7% of controls (RR = 2.62 [95% CI 2.47-2.79]), with greatest risk for autistic people without intellectual disability.
Mayes SD et al. 2012 [12]	Observational study	The sample of 1609 children 6 to 16 years of age consists of 302 children with high functioning autism (HFA, IQ greater than 80), 133 children with low functioning autism (LFA, IQ < 80), 186 typical children, and 988 children with other clinical disorders	The study shows that early intensive behavioral intervention can significantly reduce autistic symptoms and improve overall functioning and long-term outcome. Symptoms of anxiety and depression are also found in most children with autism. Children with autism had significantly higher explosive, oppositional, and aggressive scores than children with ADHD-I, anxiety, brain injury, and typical development (F > 41.0, p < .0001, Bonferroni p < .05).
Hofvander B et al. 2009 [14]	Observational study	5 patients with autistic disorder, 67 with Asperger's disorder and 50 with pervasive developmental disorder not otherwise specified	Among autistic disorder subjects: none had an anxiety disorder. Among patients with Asperger’s disorder: 34 (51%) had an anxiety disorder
Mc Dougal E et al. 2020 [16]	Observational study	Ten teachers (9 are females) of pupils with autism took part and worked in mainstream primary schools Most participants were working as a class teacher (N = 7), but two were dedicated Special Educational Needs Co -ordinators (SENCOs) with reduced class teaching responsibilities, and one was a Higher Level Teaching Assistant (HLTA)	Nearly all teachers (N = 9) described the impact of anxiety on the learning experiences of children with autism. As with sensory sensitivity, the consensus generally seemed to be that if a child is experiencing anxiety, it becomes all-encompassing so the child can’t focus on their work and they could miss out on learning. Some of the high functioning autistic children had the highest levels of anxiety Nine teachers across types of school provision described the importance of structure; a lack of structure can be anxiety inducing and therefore distracting for pupils with autism.

## Conclusions

The major limitation of this review is the paucity of available research to establish a clear relationship between childhood ASD and progression to anxiety and depression in adulthood, there remains a need for clinicians and researchers to further explore this area. Therefore, the authors propose that there should be more research in the future in studying this phenomenon. Existing studies as earlier highlighted have depicted the role of socioeconomic factors in the possible development of co-occurring mental health conditions in individuals with ASD. The behavioral symptoms seen in individuals with ASD can be attributed to the low socioeconomic status of parents, given limited access to resources for their children. Cognitive behavioral therapy reduces the development of anxiety and depression in individuals with ASD.

This review will hopefully serve as a guide for future research in this aspect, owing to the dearth of studies. More so, this will close the remaining gaps in knowledge and also give room for early intervention and prevention which would improve the quality of life of these individuals mentally, socially, and otherwise.
